# Recent Progress in Processing Functionally Graded Polymer Foams

**DOI:** 10.3390/ma13184060

**Published:** 2020-09-13

**Authors:** Supitta Suethao, Darshil U. Shah, Wirasak Smitthipong

**Affiliations:** 1 Specialized Center of Rubber and Polymer Materials in Agriculture and Industry (RPM), Department of Materials Science, Faculty of Science, Kasetsart University, Chatuchak, Bangkok 10900, Thailand; supitta.sue@gmail.com; 2 Centre for Natural Material Innovation, Department of Architecture, University of Cambridge, Cambridge CB2 1PX, UK; dus20@cam.ac.uk; 3 Office of Natural Rubber Research Program, Thailand Science Research and Innovation (TSRI), Chatuchak, Bangkok 10900, Thailand; 4 Office of Research Integration on Target–Based Natural Rubber, National Research Council of Thailand (NRCT), Chatuchak, Bangkok 10900, Thailand

**Keywords:** porous polymers, cellular materials, microstructure, property gradient, functionally graded structure

## Abstract

Polymer foams are an important class of engineering material that are finding diverse applications, including as structural parts in automotive industry, insulation in construction, core materials for sandwich composites, and cushioning in mattresses. The vast majority of these manufactured foams are homogeneous with respect to porosity and structural properties. In contrast, while cellular materials are also ubiquitous in nature, nature mostly fabricates heterogeneous foams, e.g., cellulosic plant stems like bamboo, or a human femur bone. Foams with such engineered porosity distribution (graded density structure) have useful property gradients and are referred to as functionally graded foams. Functionally graded polymer foams are one of the key emerging innovations in polymer foam technology. They allow enhancement in properties such as energy absorption, more efficient use of material, and better design for specific applications, such as helmets and tissue restorative scaffolds. Here, following an overview of key processing parameters for polymer foams, we explore recent developments in processing functionally graded polymer foams and their emerging structures and properties. Processes can be as simple as utilizing different surface materials from which the foam forms, to as complex as using microfluidics. We also highlight principal challenges that need addressing in future research, the key one being development of viable generic processes that allow (complete) control and tailoring of porosity distribution on an application-by-application basis.

## 1. Introduction

Polymer foams find a wide range of applications, including in pillows and mattresses, physical insulation, furniture, engineering materials, housing decoration, and electronic devices, etc. In comparison to metallic and inorganic (e.g., ceramic and glass) porous materials, polymeric porous materials are of interest as they are substantially lighter (because of their lower density), have lower cost, offer a wider range of compressive strengths (from elastic to flexible to semirigid to rigid), and are producible at considerably lower temperatures using a range of methods, including spray foaming [1,2,3,4,5,6,7,8,9,10]. This review will focus on foams of a polymeric nature only.

The polymer foams with supercritical fluids are attracting interest, especially for producing microporous foams. These are cellular polymer foams with approximate 10 µm in pore dimeter and 10^9^ pores per cm^3^ in pore density. These compact materials present high toughness, high impact strength, and high stiffness-to-weight ratio. In addition, polymer foams with supercritical CO_2_ do not usually require the use of harmful organic solvents. Such an advantage provides the method suitable for processing porous structures from biocompatible polymers as scaffolds for biomedical applications [11,12,13].

From a market perspective, recently reported global market values of exported polymer foam are presented in the Table 1. The exported values of polymer foam have increased from 2017 to 2018 for all types of polymer foam (based on data from the International Trade Centre (ITC) [14]).

Generally, polymer foams are porous materials that have two or more phases. In a two-phase polymer foam, the polymer matrix forms a continuous phase and the gaseous-porosity phase is composed of gas bubbles. The porous structure is produced by either a chemical or a physical blowing agent for gas bubble production in a polymer matrix. In the case of chemical blowing agents, a chemical reaction produces gas bubbles, usually through the decomposition of a chemical. By contrast, physical blowing agents are inert gases or supercritical fluids (mostly CO_2_ and N_2_), which can be dissolved into the polymer matrix during a saturation process [15,16,17,18,19,20].

Chemical blowing agents can be used for both liquid and solid polymers. Concerning liquid polymers, in particular natural latex, chemical blowing agents such as potassium oleate are used in the “Dunlop process” to manufacture rubber foams for pillows and mattresses, etc. [21,22,23,24,25]. In contrast, production with a solid polymer is performed by gas diffusion processes (induced by a chemical blowing agent) between the foam and molten polymer matrix. This type of process can be controlled by the formulation and process of polymer to be foamed [26].

## 2. Polymer Foaming Process

Normally, the process of polymer foam production with a physical blowing agent is divided into two principal steps. First, the polymer matrix is saturated with a physical blowing agent (gas or supercritical fluid) at constant conditions. Next, the supersaturated state is brought about by phase separation, induced either by rapidly increasing temperature or reducing pressure, for generating gas bubbles, and therefore cells, inside the polymer matrix [27,28,29,30]. The cells grow to reach the point that the viscosity of the polymer matrix is increased corresponding to the force opposing the expansion of the foam until it becomes sufficiently high [31]. The foam density depends on the gas loading or the gas fraction in the polymer matrix, the cell size and distribution count on the cell nucleation process, and the expansion process [32].

**Figure 1:**
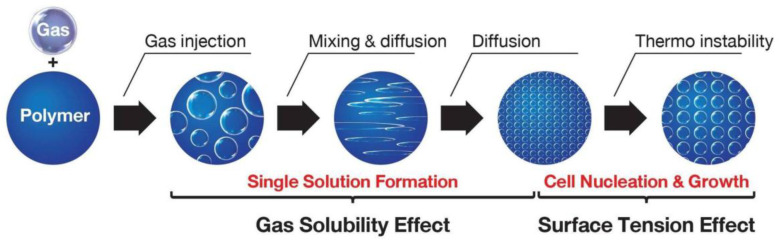
Overview of the microcellular foaming process.

In the case of plastic foam (or non-rubber foams), there are three basic processing steps: (1) polymer/blowing agent solution mixing, (2) microcellular nucleation, and (3) cell growth and density stabilization. The first step of single-phase polymer/blowing agent solution mixing is formed by saturating the polymer with the blowing agent under certain conditions. The saturation point is determined by the solubility limit of the blowing agent in the polymer, while the time required for the solution formation is determined by the rate of diffusion of the blowing agent into the polymer matrix. Microcellular nucleation (Figure 1) is controlled by inducing a thermodynamic instability in the single-phase solution. This is usually succeeded by drastically reducing the solubility of the gas in solution by operating the pressure and/or temperature of the mixture [33,34,35,36,37,38,39,40]. Since the separation of the polymer and gas phases is thermodynamically more favorable, the resulting supersaturated mixture becomes the driving condition for the nucleation of numerous microcells. Continuous microcellular processing typically utilizes a rapid pressure drop to nucleate bubbles. This stage is very crucial to the overall process, because it dictates the cell morphology of the material and its resulting properties. Therefore, solubility as a function of pressure is important for the development of the process. The final stage in the production of microcellular plastics is cell growth. After cell nucleation has occurred, any available gas diffuses into the cell and increases its size, thereby reducing the density of the polymer matrix. Generally, the growth of cell depends on the time allowed for the cells to expand, the system temperature, the amount of gas available, the processing pressure, and the viscoelastic properties of the polymer/gas solution [41,42,43,44,45].

Typical foaming processes can be classified into batch foaming, and extrusion and injection molding. The batch foaming process has lower process temperatures than those needed in other processes; this causes an increasing of the CO_2_ solubility in amorphous polymers, resulting in higher cell densities and smaller cell sizes [46]. Such foam characteristics, i.e., cell size and cell density, affect directly the mechanical properties of polymer foam [47]. Generally, the batch foaming process is utilized to amorphous polymers, which starts from the rubbery state at the saturation condition. On the other hand, the semicrystalline polymers possess the non-uniform cell structure because of the inability of the polymer/blowing agent formation in the crystalline structure. This type of crystalline structure of the polymer needs to be destroyed before foaming due to the melting point reduction of the polymer using a co-solvent [47]. 

Concerning the process of polymer foaming with a physical blowing agent, the porous structure of the polymer/gas (fluid) system depends on the important parameters below [48,49]:

(i) the degree of crystallinity of the polymer matrix,
(ii) the amount of the dissolved gas (fluid),
(iii) the degree of saturation of gas in the polymer,
(iv) the interfacial energy of polymer/gas (fluid), and
(v) the plasticization profile of the polymer/gas system (i.e., the melting point and the glass transition temperature, *T_g_*, of polymer matrix).

Interestingly, among conventional polymer foams, the different cell sizes (graded density structure) attract far more attention compared with a uniform cell structure, because this type of functionally graded structure exhibits better mechanical properties compared with conventional foams [50,51]. However, fabrication of such functionally graded foams is complicated. Generally, the functionally graded structure of polymer foam can be obtained by foaming process, nanofiller, blowing agent, or polymer composition, etc. In recent years, various graded cellular materials have attracted interest [52,53]. The polyethylene foam with density gradient improved the mechanical properties due to the change of deformation mode [54]. The polyurethane honeycombs with four density gradients were modified from the uniform density equivalent using different parameters: fused filament fabrication 3D printing, density grading, energy absorbing, and damping profiles [55]. Several types of graded foam are produced for functional applications, such as impact strength, acoustic capabilities, energy absorption, etc. These new graded foams (polyurethane foam, acrylonitrile butadiene styrene foam, polyethylene foam, polypropylene foam, polylactic acid foam, and polymethyl methacrylate foam) are investigated in the structure–property relationship [56,57,58,59,60,61].

The main aims of this review paper focus on the importance of structure-property-processing relations in polymer foams. In particular, recent polymer foams with cell size gradients or functionally graded foams are of interest. Functionally graded foams are foams that incorporate various cell sizes in the same material and therefore possess a structure with a “cell size gradient”. This structure could be mimicked from structural materials found in nature, such as bones and bamboo. These types of foams could be useful for tailor-made material products with functional properties ranging from thermal insulation, to high stiffness or strength at low weight, to buoyancy, and impact resistance [62,63,64,65]. Consequently, cellular structure with cell size gradients (different cell sizes) has received interest from both academic and industrial sectors.

## 3. Thermodynamic Aspects and Computer Modeling of Polymer Foam Processing

**Figure 2:**
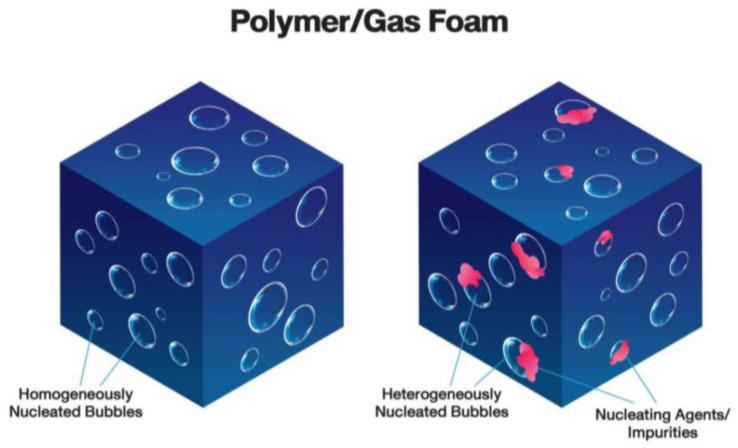
Schematic of homogeneous and heterogeneous nucleation in a polymer/gas system.

The mechanism of gas bubble nucleation inside the polymer matrix for the relevant foaming method is very complex, governed by multiple phenomena, including interfacial energy of polymer/gas system. The creation of gas nuclei can be related to either homogeneous or heterogeneous nucleation (Figure 2). Homogeneous nucleation possesses the spontaneous generating of gas molecules in the polymer matrix; on the other hand, heterogeneous nucleation exhibits the gas nuclei on the boundaries of two phases (polymer and another material like filler) [66,67,68,69,70]. In the case of plastic foaming without use of a chemical agent, bubble nucleation is often assumed to be homogeneous. However, in the process of rubber foaming with a chemical agent and filler, both homogeneous and heterogeneous nucleation occur.

Nucleation refers to the initial stage of gas bubble formation from the initial polymer matrix. In this step, a gas bubble has to conquer the Gibb’s free energy before the bubble can grow to an optimum scale. This step is explained by classical nucleation theory: the difference of Gibb’s free energy of the polymer/gas system can be expressed as the sum of gain in the Gibb’s free energy related to the formation of interface. Generally, in an isothermal system at chemical equilibrium, the difference of Gibb’s free energy (ΔG) corresponds to the formation of polymer/gas system, which is expressed by the equation [31]:

(1) $$\Delta G=\left(-4\pi r^{3}/3\right)\Delta P+4\pi r^{2}\gamma ,$$

where r is the radius of spherical cluster, γ is the interfacial energy between gas and polymer, and ΔP is the difference of pressure. The next equation is obtained and relates to homogeneous nucleation, as ΔG is plotted against cluster size with a maximum at a critical radius, $r_{c}$, is obtained:

(2) $$d\Delta G/dr=0\;\mathrm{thus}\;r_{c}=2\gamma /\Delta P,$$

The maximum value of $\Delta G^{*}$ for homogeneous nucleation is derived by substituting Equation (2) into Equation (1) as [71]:

(3) $$\Delta G^{*}=16\pi \gamma^{3}/3\Delta P^{2},$$

Decreasing the interfacial energy, or increasing the difference of pressure, results in increasing the nucleation rate. However, the interfacial energy of the polymer/gas system is complicated to measure. Normally, such interfacial energy is calculated corresponding to the surface energy of each material at equilibrium [31,72]; the limitation of this theory relates rather to the determination of this parameter.

The presence of fillers, suspension of chemical agents, impurities, or another material in the system is the cause of heterogeneous nucleation. Generally, the presence of particles or impurities decreases the Gibb’s free energy Δ*G* and involves a reduction factor *f* as:

$$\Delta G_{het}=\Delta G_{hom}\left(f_{\left(m,w\right)}/2\right),$$

(4) $$m=\cos\theta =\left(\gamma_{13}-\gamma_{23}\right)/\gamma_{12}\;\mathrm{and}\;w=R/r_{cr}\;(\mathrm{relative}\;\mathrm{curvature}),$$

where R is the radius of particles; $\gamma_{13}$, $\gamma_{23}$, and $\gamma_{12}$ are the interfacial energies of polymer/particle, gas/particle, and polymer/gas, respectively; and θ is the contact angle between the cell, polymer, and particles.

**Figure 3:**
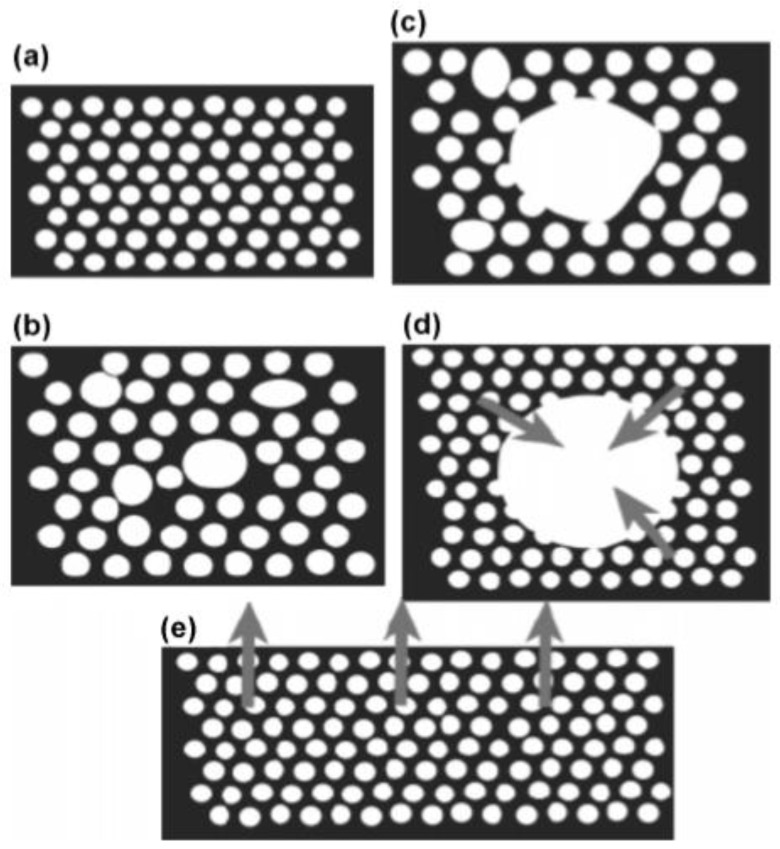
Illustration of cell formation at (**a**) *T_nuc_* << *T_g_*; (**b**) *T_nuc_* < *T_g_*; (**c**) *T_nuc_* = *T_g_*; (**d**) *T_nuc_* > *T_g_* in polymer; and (**e**) *T_nuc_* > *T_g_* near surfaces [73]. (*T_nuc_*—foaming nucleation temperature, *T_g_*—glass transition temperature.) Adapted from [73], with permission from © 2005 American Chemical Society.

The type of nucleating agents affects the nucleation process of polymer foam, which can be explained by continuum conservation models. Concerning the polymer melt, thermodynamic fluctuations allow nucleus growth due to the surface and viscous forces. When the pressure inside the cell decreases, the gas concentration at the cell surface also decreases [15,66]. In the batch foaming process, the cell growth certainly depends on the temperature process. Based on classical nucleation theory, when the foaming nucleation temperature (*T_nuc_*) decreases, the formation of smaller cells can occur. If *T_nuc_* is below the glass transition temperature (*T_g_*) of high viscosity polymer matrix, nanocell structures can appear. On the other hand, microcell structures appear when the *T_nuc_* closes to the *T_g_* of the polymer matrix (Figure 3).

Recently, flexible polymers like compressible elastomers with different densities (graded materials) have been described by a worm-like chain model [74]. A self-consistent field theory (SCFT) was developed using thermodynamic calculations of the system based on Helmholtz free energy. There were two types of length scales relevant for a flexible polymer chain: polymer length L and persistence Lp. The ratio of polymer length/persistence (L/Lp) ratio was found to be L/Lp << 1 for rod-like elastomer structures, while L/Lp >> 1 was proposed for coil-like elastomer structures. This combination of thermodynamic and computational modeling paves the way for a new foam material.

Foam processing computational models are useful to predict and estimate the properties of polymer foams. These models are often related to a finite element (FE) method. These empirical models employ time- or temperature-dependent density related to the nucleation and growth of bubbles in the polymer foam. The continuum-level model applies a description of homogeneous nucleation through the density model but does not include the gas model; this type of model has been developed to explore optimum properties of liquid phase/gas bubbles during the self-expansion process [75,76]. 

Foam rheological property measurements are complicated to carry out, since the foam microstructure usually changes. Thus, the viscosity is separated into two parts dependent on (1) continuous-phase polymer properties and (2) gas bubble volume fraction: these two phases are quite different [77]. The component mass fractions and densities can be utilized to determine the gas volume fraction using the density model of polymer foam. Moreover, the gas volume fraction components of foam heat capacity and thermal conductivity, which can be utilized for the energy equation. The foam heat capacity is calculated by the mixture theory for polymer/gas system [78]. The effect of liquid vaporization can be defined from the density evolution and the mass fraction of polymer/gas system [76,79].

There has been increasing recent interest in applying the concept of graded cellular materials to polymers in order to improve their mechanical properties. Such cellular polymers exhibit a gradient in their properties, for example, cell size/cell density, cell distribution, mechanical properties, etc. [54,62]. Therefore, it is worthy to explore the processing and mechanisms of graded cellular polymers, which can be used to control desirable properties and behaviors. For example, researchers investigated the behaviors of voronoi-type density gradient foams using the finite element (FE) method [80]. The results obtained show that the energy absorption is linked to the profiles of graded cell distribution. The FE simulation can be also utilized to study the effect of temperature gradient on the properties of graded foam [81]. Moreover, the density-graded models can be investigated for the deformation pattern and energy absorption capacity of the resulting materials produced using a temperature gradient. The latest advances of energy absorption (or impact resistance) for functionally graded foams relate to density and temperature gradients during foam processing. For example, an increasing temperature gradient leads to the reducing of energy absorption capacity in functionally graded foam [80,81]. 

## 4. Recent Processes to Produce Functionally Graded Foams

**Figure 4:**
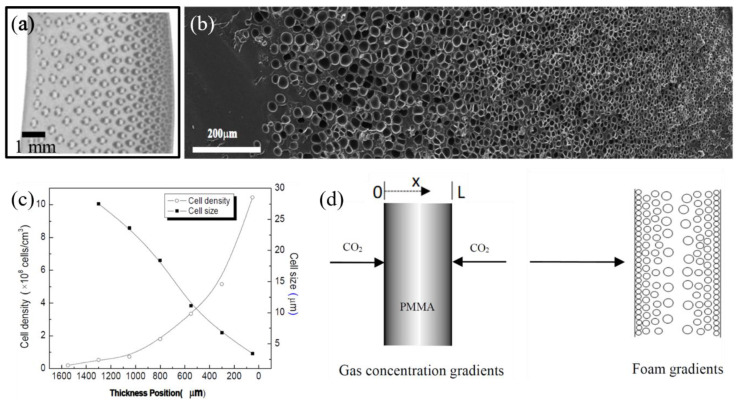
The functionally graded structure of bamboo (**a**) is comparable to the graded microporous foams produced by Yuan et al. [86] (**b**), in which cell size and density are correlated to the location (**b**,**c**), much like in bamboo (**a**). (**d**) The foam gradients are a result of gas concentration gradients during processing. Images (**b**–**d**) by Yuan et al. [86].

New processes have been developed to produce polymer foams with gradients in cell size (gradient density) and properties (functionally graded) [82,83,84,85]. A gradient structure imparts the foam with an asymmetric structure of cell size (Figure 4), with cell size at one end being smaller (and denser). This structure provides superior properties, such as mechanical properties, and proposes new functionalities for various applications, including in sound absorption and protective equipment (helmets). 

An example process of foaming with cell size gradient was presented in [86], where polymethyl methacrylate (PMMA) absorbed CO_2_ at 28 MPa and 50 °C for 1 h in order to form the PMMA foam. Figure 4 shows that the cell size at the near surface is smaller than the cell size faraway from surface. The effect of high CO_2_ concentration in the surface increases cell density and reduces cell size.

**Figure 5:**
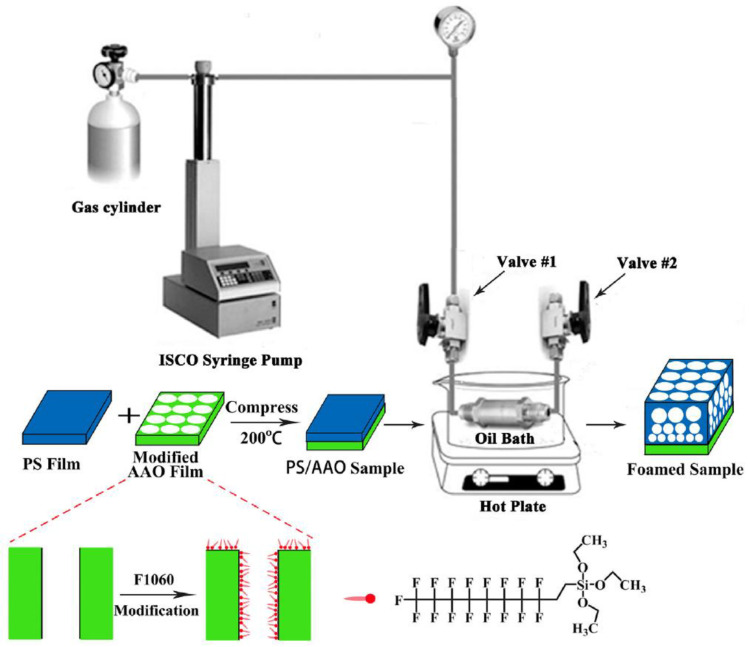
Using a conventional batch foaming method for the processing of graded polymer foams [82]. Adapted from [82], with permission from © 2016 Elsevier.

**Figure 6:**
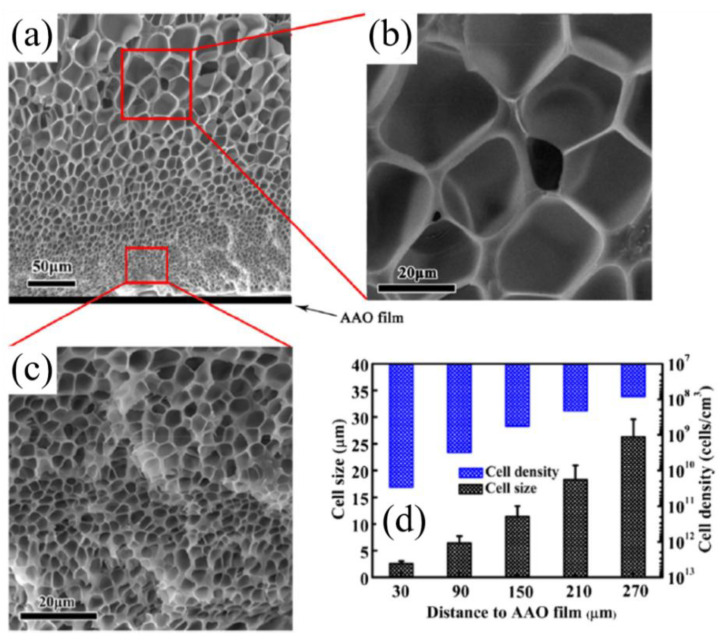
(**a**–**c**) SEM images of graded polystyrene (PS) foam structure; (**d**) cell size and density at different distance from the surface [82]. Adapted from [82], with permission from © 2016 Elsevier.

Yet another process that produces gradient density polymer foams is when aluminum oxide (AAO) film is used as a surface material for foam preparation [82]. Silane fluoride is used as an agent to change the surface of the substrate. Polystyrene, polymethyl methacrylate, and polyacrylate may be used as the polymer and CO_2_ as the blowing agent. For the method of foam preparation (Figure 5), AAO film is modified by fluorinated silane using an impregnation method. The polymer plate is placed on the AAO film and compressed to form a composite structure. The foaming process uses supercritical CO_2_ as a blowing agent at 13.8 MPa and 100 °C for 12 h, permitting CO_2_ diffusion and reaching an equilibrium state. Figure 6 presents the morphology of polystyrene (PS) foam on anodized aluminum oxide (AAO) film from an SEM image. This method is also successful in producing foam with cell size gradients. The results (Figure 6d) show that near AAO film surface, the cell size is small, while cell density is high. Further away from AAO surface, the cell size is bigger, while cell density is decreased [82].

Gradient density foams with low-density polyethylene (LDPE) have also been reported in literature. Azodicarbonamide is used as a chemical blowing agent, and silicone rubber sheet (SRS) is used as a contact material during the foaming of the polymer. ZnO is used to reduce high-temperature decomposition of azodicarbonamide, and stearic acid is used as an extrusion processing aid. All materials were mixed using a twin-screw extruder, and then LDPE foams were injected inside a mold at temperatures during 200 and 240 °C [26]. The construction of outer solid skin is formed by gas diffusion from the matrix to the surface of mold. The results show that the internal pressure of foam increases when the amount of azodicarbonamide is increased; this consequently affects final density by inducing significant gas absorption in silicone at the surface. X–ray visualization revealed that the properties of foams produced with or without SRS were the same results. However, foams produced with SRS showed the construction of a dense skin of SRS between 1800 and 2990 s, whereas the conventional foam appears to remain free of a solid skin. Increasing foaming temperature and maximum internal pressure induce an increase in the solid skin thickness.

LDPE foams produced without SRS presented pores reaching right to the surface of the foam. By contrast, a solid skin at the foam surface with significant thickness is observed when SRS is applied into the mold at the foaming preparation. Based on this new process, two types of foam structure are found: solid skin and porous core. The tensile stress–strain behavior of the skin, core, and structural foam are distinct. From a mechanical properties point of view, the core has the lowest modulus and strength, and the solid skin has the highest modulus and strength, whereas values of the structural foam remain intermediate to the skin and core. The presented process allows the design of gradient density foam structure and related properties [26].

Another interesting example of graded foam is a monodisperse polystyrene (PS) foam [87]: this type of foam can program the pore size and density using different pressures during the foaming process. Concerning this method, one can represent a cell size gradient in the PS foam using the gas pressure variation, in particular, the big cell size of PS foam can be obtained by increasing the gas pressure in the preparation method. 

So far we have reviewed functionally graded polymer foams based on PMMA, LDPE, and PS. A range of other thermoplastic polymers have been utilized to produce foams with a controlled structure, including polypropylene (PP) and polycaprolactone (PCL). In a study by Yang et al., hollow molecular-sieve (MS) particles were used as a nucleating agent in supercritical carbon dioxide (scCO_2_) for polypropylene (PP) foams. In this study, the PP pellets and MS particles were mixed using a twin-screw extruder before they were pressed into sheets with a thickness of 1 mm. They were then foamed inside an autoclave using CO_2_ as a blowing agent under pressure of 20 MPa and temperature of 154 °C for 2 h [88]. Yang et al. [88] found that addition of MS particles substantially decreased the distribution in cell sizes of the PP foam, with the foam cell density increasing by an order of magnitude, and doubled the tensile strength. In another study, Llewelyn et al. used a hybrid foaming method by utilizing a physical blowing agent (super critical nitrogen) and a chemical blowing agent to produce polypropylene (PP) foam, with and without talc filler, by low-pressure foam-injection molding (FIM). Through a hybrid foaming method with low pressure (FIM), foams with high cell density and superior homogeneous cell structure were produced [89]. To produce inhomogeneous foams, using chemical blowing was most effective, as a larger skin wall thickness was obtained. Using a thermally induced phase-separation method, Onder et al. produced polycaprolactone (PCL) foams [90]. The PCL solutions were prepared in the tetrahydrofuran/methanol (THF/MeOH) solvent system by slowly heating to 55 °C in a water bath. The homogeneous polymer solutions were quenched at low temperature for 12 h. The PCL foams were warmed up to room temperature and thereafter dried by vacuum drying. PCL foams with larger pores were obtained at lower PCL concentration, lower THF content, and a higher quench temperature [90].

Apart from foams based on thermoplastic polymers, thermoset polymers and elastomers have also been successfully used to produce foams with controlled structure (and therefore properties). Song et al. [89] mixed a biobased epoxy resin (Greenpoxy 56) and an amine-based hardener using a hand-held mixer for 20 min in order to produce an air-in-resin liquid foam. The biobased polymer foams were formed in self-standing tubes. The porosity of biobased polymer foams was increased from 71% to 85% by heating the air-in-resin liquid foam during the curing step [91]. The compressive modulus and compressive strength of the polymer foams were significantly reduced as a result of an increase in porosity. Vahidifar et al. mixed natural rubber (NR) compounds at room temperature using a two-roll mill to produce an elastomeric foam. The one-step foaming process for the natural rubber/carbon black (NR/CB) foam production was performed by compression molding using an electrically heated press at temperature of 160 °C and pressure of 50 kPa for 30 min. Cell density of NR/CB foam was increased around 14 times by increasing the CB content at the same foam density. The morphology of NR/CB foams was divided into three layers: outer (no cells), middle (indeterminate cells), and inner (circular cells) [92].

## 5. Conclusions and Future Research Outlook

Functionally graded polymer foams are an emerging innovation in polymer foam technology. Their combination of light weight with efficient use of material and enhanced functional properties help the design of multifunctional products. For example, the graded structure may enable efficient performance in low-energy impact as well as high-energy impact applications. Such functionally graded polymer foams may find diverse applications, such as in tissue bioengineering, protective gear (helmets), engineering structures (construction core materials and automotive car bumpers), and filtration and insulation.

**Figure 7:**
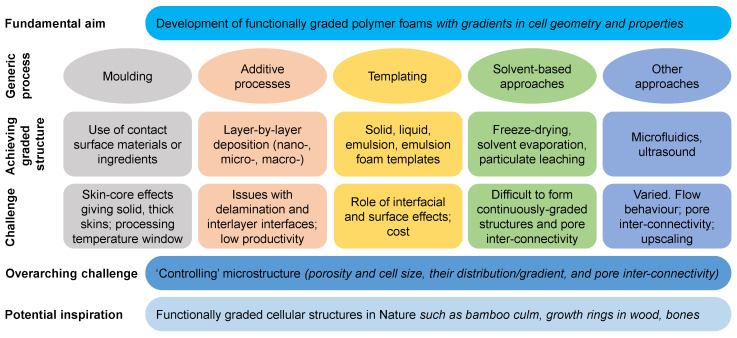
Synthesis of various approaches to process functionally graded polymer foams, and their associated advantages and challenges.

A variety of approaches have been employed by scientists to produce functionally graded polymer foams, synthesized in Figure 7. A number of researchers have explored molding processes, such as (reaction) injection or compression molding, and have employed the use of specific contact surface materials (such as aluminum oxide film or silicone rubber sheet) or ingredients (such as supercritical CO_2_ as a blowing agent). These have enabled the production of foams with gradients in porosity, cell size, and properties, sometimes with a skin-core structure. Additive processes, such as 3D printing and, more commonly, layer-by-layer lamination techniques (e.g., with thermal bonding), have also been developed to produce functionally graded polymer foams. Each layer may have a distinct pore and cell size, and the layers can range from nano- to micro- to macroscale. However, in such additive manufactured foams, the presence of interlayer interfaces, which are regions of stress transfer and stress concentration, increases issues with delamination and crack propagation. Templating routes have also been examined using solid, liquid, and emulsion foam templates. Solvent-based approaches (including particulate leaching and freeze drying) have also been explored. Mixed success has been achieved using these for functionally graded polymer foam manufacture. While some approaches may enable achieving versatile structures, producing nanoporous to macroporous structures, the processes may hinder the formation of continuously graded structures and pore interconnectivity (open cell and closed cell structures). A number of other processes have been also developed in literature, including those based on microfluidics and the use of ultrasound to produce heterogeneous polymer foams.

The overarching and biggest challenge is control on microstructure. This includes control on porosity and cell size, the gradient (qualitative such as continuously graded or discretely graded structure or skin-core structures, but also more tailored quantitative), and pore interconnectivity (open-cell or closed-cell or mixed and the extent to which they are tailorable). There is still some way to go in understanding these processing-structure-property relations, let alone controlling them, and developing viable generic processes for tailorable functionally graded polymeric foams.

Indeed, better understanding of processing-structure-property relations will require multidisciplinary approaches, including empirical process science and polymer foam technology, but also the physical thermodynamics and chemical kinetics behind the formation of functionally graded polymer foam microstructures, as well as process simulation and computer modeling. The latter may offer more time-saving, cost-effective methods for obtaining insights and optimized routes to fabrication and rapid prototyping for bespoke products (particularly in bioengineering, such as scaffold design and 3D printing for individual patients).

Yet another discipline that may offer insights is the life/biological sciences. An architectural engineering marvel is the Eiffel Tower, which was designed by Gustave Eiffel on the principles of material minimization, through inspirations from the femur bone: a natural, functionally graded cellular structure. Biomimetics and bioinspiration are important sources of design concepts. The intelligent design of functionally graded polymer foams following designs in nature can unveil important insights on structure–property relations. For example, the selection of the polymers—based on their chain length and molecular weight, functional groups and species, and so on)—and how the polymer chains interact with each other will have a notable impact on the morphologies and properties of the foams produced. For instance, in wood and plant stems, other natural functionally graded foam structures, the complex self-assembly and interaction of various polymer species (cellulose, hemicellulose, lignin, pectin, and so on) lead to a beautiful hierarchical cellular structure, making it a model functionally graded material [93,94,95,96,97,98,99,100]. Indeed, the minute changes in the polymer species, and their interactions and formations, lead to a wide variety of wood species in nature with a range of functional properties, including densities, strengths, and hardness [93,101,102,103,104,105,106,107,108].

Moreover, bioinspiration can help produce multifunctional products for specific applications, such as scaffolds for interfacial tissue engineering (e.g., cartilage-bone scaffolds with an order of magnitude difference in modulus between the cartilage and the bone), and protective foam shell of helmets inspired from the functionally graded structures of sheep horn and horn core trabecular bone of bighorn sheep rams. Such studies may also later fuel the design of hierarchical (multiscale porosity distribution) functionally graded polymer foams and composite, fiber-reinforced or polymer-blended, functionally graded polymer foams, taking bamboo as an inspiration, for instance. Furthermore, rather than being based on petrochemical-derivative polymers, exploration of bio-derivative polymers for the fabrication of functionally graded polymer foams may enable improvement in biocompatibility (for bioengineering) and biodegradability (for better end-of-life options) [109,110,111,112,113,114,115].

There have been important technological, process-related advancements in functionally graded foams over the past couple of decades, and further understanding and control of the process is key to their inevitable utilization in functional products.

## Figures and Tables

**Table 1:** Values of the polymer foam exported to the world during 2017–2018 [14].

Types of Polymer Foam	Exported Value in Million USD (% of Total)			
	2017		2018	
Polystyrene foam	1.276	(10.3%)	1.339	(9.9%)
Polyvinyl chloride foam	1.799	(14.5%)	2.001	(14.9%)
Polyurethanes foam	3.860	(31.1%)	4.167	(30.9%)
Other plastic foams	4.426	(35.7%)	4.852	(36.0%)
Rubber foams	1.053	(8.5%)	1.110	(8.2%)
